# In Silico Characterization of Two Human Pegivirus Proteins Highlights Similarities with Hepatitis C Virus and Possible Therapeutic Repurposing

**DOI:** 10.3390/v18020261

**Published:** 2026-02-19

**Authors:** Kaleigh M. Copenhaver, Barbara A. Hanson, Joshua J. Ziarek, Igor J. Koralnik

**Affiliations:** 1 Department of Neurology, Northwestern University Feinberg School of Medicine, Chicago, IL 60611, USA; kaleigh.copenhaver@northwestern.edu (K.M.C.); barbara.hanson@northwestern.edu (B.A.H.); 2 Department of Pharmacology, Northwestern University Feinberg School of Medicine, Chicago, IL 60611, USA; joshua.ziarek@northwestern.edu

**Keywords:** drug repurposing, Hepatitis C virus (HCV), Human pegivirus (HPgV), Parkinson’s disease

## Abstract

Human Pegivirus (HPgV) is an understudied flavivirus that is highly prevalent and often persists in the blood and tissues of humans. HPgV-infected brain tissue from individuals with Parkinson’s disease has shown significant transcriptomic and immune signaling differences compared to non-infected Parkinson’s brains. The HPgV genome is similar to Hepatitis C Virus (HCV), a well-characterized flavivirus with multiple approved small-molecule therapeutics. Here, we used HCV crystal structures to create homology models for two HPgV non-structural (NS) proteins, the serine protease (NS3) and the RNA-dependent RNA polymerase (NS5B), and performed molecular dynamic simulations. HCV and HPgV proteins had minimal structural differences, as seen by the Root Mean Square Deviation (RMSD) difference between NS3 (1.00 Å) and NS5B (1.26 Å). FDA-approved small molecules were then docked in silico to the NS3 and NS5B subunits of HCV and HPgV. HCV had weak to moderate correlated docking scores with HPgV NS3 (R^2^ = 0.21, *p* < 0.001) and NS5B (R^2^ = 0.58, *p* < 0.001). The predicted protein–ligand interactions showed potential binding between HCV antivirals and conserved residues of HPgV, including the catalytic triad for NS3 or the GDD motif for NS5B. Together, these results provide structural insights for key HPgV proteins and highlight possibilities for therapeutic repurposing of HCV antivirals.

## 1. Introduction

Human Pegivirus (HPgV) is an understudied flavivirus with striking sequence similarity and genomic organization to Hepatitis C Virus (HCV) [1]. Up to 20% of individuals infected with HCV are suspected to have a coinciding HPgV infection, which rises to 37% in people co-infected with HCV and HIV [2,3,4]. HPgV infections are common and typically subclinical, but HPgV has also been detected in central nervous system (CNS) tissues in individuals with CNS diseases suggesting a possible relationship [5,6,7,8]. We recently found an association between HPgV infection and significant transcriptomic and immune signaling changes in brain tissues from HPgV-infected Parkinson’s disease patients [9].

**Figure 1:**
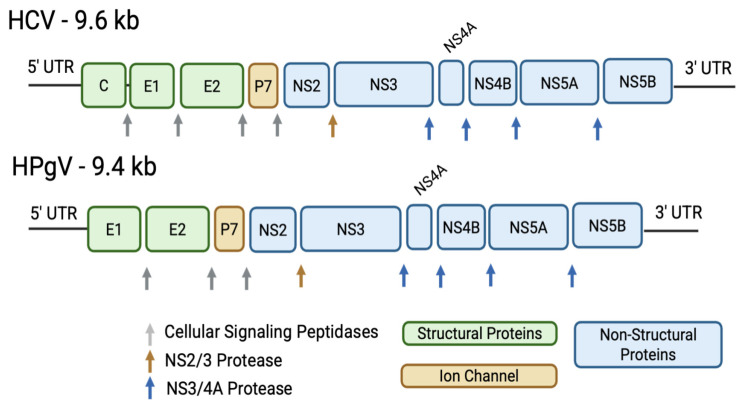
Comparison of HCV and HPgV Genome organization. HCV and HPgV structural and non-structural genome organization. Arrows represent cleavage sites by cellular signaling peptidases, NS2/3 protease, or NS3/4A protease.

Both HCV and HPgV genomes encode a single polyprotein that is cleaved into structural and non-structural (NS) proteins. In HCV, the structural proteins are Core, envelope proteins E1 and E2, followed by seven NS proteins (P7, NS2, NS3, NS4A, NS4B, NS5A, and NS5B) (Figure 1) [2,4,10,11]. HPgV encodes homologous structural proteins (E1, E2) and seven NS proteins (P7, NS2, NS3, NS4A, NS4B, NS5A, and NS5B). While several HPgV NS proteins remain incompletely characterized, experimental studies have demonstrated that HPgV NS3 functions as an active serine protease and that NS4A serves as a protease cofactor [12,13,14]. NS4B is a small hydrophobic protein thought to contribute to the formation of virus-induced membrane rearrangements, and NS5A is a phosphorylated protein implicated in RNA replication [10,11].

Several direct-acting antivirals (DAAs) target the HCV NS3/4A protease, NS5A, or NS5B polymerase, but no therapeutics are approved for HPgV (Table 1) [14,15]. Recent studies investigating HPgV prevalence among HCV-HPgV co-infected individuals treated with HCV DAAs showed promising results, particularly when combined with pegylated interferon [16]. Here, we used in silico approaches to model HPgV NS3/4A and NS5B structures and to assess whether approved HCV DAAs show predicted binding to these essential HPgV proteins.

## 2. Materials and Methods

### 2.1. HCV and HPgV Consensus Sequence Alignment

For generation of the NS5B consensus sequence, nineteen full-length HPgV polyprotein sequences were selected to represent genotype diversity, including Genotype 1 (AB013500.1, U36380.1); Genotype 2a/b (AF104403.1, AY196904.1, D90600.1, KP259281.1, LT009485.1, LT009494.1, U44402.1, U45966.1, U63715.1); Genotype 3 (AB008335.1, D87713.1, D90601.1, U86151.1); Genotype 4 (AB018667.1, AB021287.1); Genotype 5 (AY949771.1); Genotype 6 (AB003292.1); and Genotype 7 (HQ331233.1, HQ331234.1, HQ331235.1). Sequences were chosen to provide broad genotype representation and were previously used for phylogenetic analysis, as described in Hanson et al. [9]

NS3 consensus sequence comprised all full-length HPgV polyprotein sequences available on NCBI virus on the date of accession (*n* = 63; NCBI accession numbers: AHA61262.1, AXR98540.1, QDK56780.1, QKY59672.1, QKY59674.1, QKY59676.1, AXR98538.1, AXR98539.1, AXR98541.1, AXR98542.1, AXR98543.1, AXR98544.1, QKY59673.1, QKY59675.1, QTH79642.1, UOW86610.1, UOW86611.1, UOW86612.1, UOW86616.1, UOW86617.1, CVH74186.1, UOW86613.1, UOW86614.1, UOW86615.1, AHA61261.1, AHA61263.1, AXN77727.1, AYD60143.1, CVH74177.1, CVH74178.1, CVH74179.1, CVH74180.1, CVH74181.1, CVH74182.1, CVH74184.1, CVH74185.1, CVH74187.1, CVH74188.1, CVH74189.1, QGN67998.1, UJH93633.1, UJH93634.1, UJH93635.1, UJH93636.1, UJH93637.1, UJH93638.1, UJH93639.1, UJH93640.1, UJH93641.1, UJH93642.1, UJH93643.1, UJH93644.1, UJH93645.1, UJH93646.1, UJH93647.1, UJH93648.1, UJH93649.1, UNY86313.1, UNY86314.1, UNY86315.1, USL94632.1, CVH74183.1; accessed 14 July 2025) [9,17]. The NS3 sequence genotypes were a mix of HPgV-1, 2, 3 and 5, although many were not annotated with a genotype sequence and were aligned using the EMBL-EBI Job Dispatcher and the MUltiple Sequence Comparison by Log-Expectation (MUSCLE) framework [18]. A consensus sequence available in the supplemental text was generated from the multiple alignment using EMBOSS Cons [19]. All NS5B sequences were conserved for the active site Glycine, Aspartate, Aspartate (GDD) residues at positions 307–309; similarly, all NS3 sequences were conserved at amino acids HIS56, ASP79, and SER137, which comprise the active site residues for catalytic function.

Non-structural protein locations were estimated based on previously published literature [2,3,4,8,9].The similarity between HPgV consensus protein sequences was compared to HCV sequences using NCBI protein–protein BLAST parameters [20].

### 2.2. HPgV Homology Structures

SWISS-MODEL was used to identify homologous structural templates using BLAST (v2.17.0) and HHblits (v3) [21,22,23]. HPgV consensus sequences were aligned to the top candidate HCV template determined by SWISS-MODEL, and 3D homology models were generated. HCV templates were ranked by sequence similarity and the Global Model Quality Estimate (GMQE), which integrates sequence identity, alignment coverage, and template quality into a single predictive score (range 0–1). The highest GMQE template was selected for model building; templates with GMQE < 0.7 were excluded from further analysis.

Model geometry was evaluated by the percentage of residues in favored and allowed regions of the Ramachandran plots produced by SWISS-MODEL. HPgV SWISS-MODEL structures were aligned to experimentally determined HCV structures (NS3/4A: Protein Database accession [PDB] 4A92; NS5B: PDB 1YUY), and backbone RMSD values were calculated using PyMOL’s super command with no outlier rejection [24,25]. To independently assess model robustness, AlphaFold2 was also used to generate HPgV NS3/4A and NS5B structures from the same consensus sequences [26]. AlphaFold models were aligned to the HPgV SWISS-MODEL structures and trimmed RMSD values were determined.

### 2.3. Preparing HPgV Proteins for Docking

Stable HPgV NS3/4A and NS5B homology models from SWISS-MODEL and AlphaFold were prepared for docking with OpenBabel (version 3-1-1) [27]. Hydrogens were added according to the expected protonation state at physiological pH, and the structures were converted to a .pdbqt format. HCV NS3/4A (PDB: 4A92) and NS5B (PDB: 1YUY) were prepared similarly, but experimental ligands required for stabilization were removed prior to hydrogen addition. Docking grids were centered on conserved catalytic residues. For NS3/4A, a 15 × 15 × 15 Å grid box was positioned around the catalytic triad (HCV: HIS57, ASP81, SER139: grid center: x = −7.5, y = 12.5, z = 4.0; HPgV: HIS56, ASP79, SER137: SWISS-MODEL grid center: x = −9.0, y = 12.0, z = 2.5; AlphaFold x = −0.5, −27.8, 3.6). For NS5B, a similar box size was centered around the GDD motif ((HCV: G317–D318–D319: x = 1.6, y = 77.6, z = 71.2, HPgV: G307–D308–D309: SWISS-MODEL x = 1.6, y = 77.6, z = 71.2; AlphaFold x = 2.6, y = 5.9, z = −1.2).

### 2.4. Ligand Preparation

DrugBank was accessed for the complete list of FDA-approved small molecules as two-dimensional structures (*n* = 2203; date accessed August 2025) [28]. Structures were converted to three dimensions and protonated to expected state at physiological pH using OpenBabel (v3-1-1) [27].

### 2.5. Molecular Docking and Analysis of Top Compounds

AutoDock Vina 1.1.2 (The Scripps Research Institute, La Jolla, CA, USA) was used for molecular docking (exhaustiveness = 50, num_modes = 25) [29,30]. Ligands were ranked for each NS protein by average binding affinity scores. Pearson’s correlation was used to evaluate the similarity of docking scores between HCV and HPgV proteins across all small molecules. A Wilcoxon-Rank sum test assessed the difference in mean docking scores for DAAs between HCV and HPgV. Residue interactions near the catalytic regions for the top 10 compounds for each HPgV protein were analyzed in PyMol (Version 3.1) [31]. The most favorable protein–ligand conformations based on affinity score and visualization were input into LigPlot (v.2.3) to generate 2D ligand–protein interaction diagrams [32].

### 2.6. Molecular Dynamics

The HPgV NS3/4A and NS5B homology models without ligands underwent molecular dynamics (MD) simulations using GROMACS (Version 5.0.4) [33,34,35]. Proteins were centered in a dodecahedral periodic box with a minimum distance of 1.0 nm between box boundaries. The system was solvated with TIP3P water, and counterions were added to neutralize the total charge. The CHARMM36m force field was used for all simulations. Energy minimization was performed using the steepest descent algorithm to remove steric clashes. The system was equilibrated in two phases: (1) NVT equilibration for 100 ps (300 K), followed by (2) NPT equilibration for 100 ps (atmospheric pressure). Position restraints were applied to heavy protein atoms during equilibration. Production MD simulations were then conducted for 100 ns with a 2 fs timestep. Trajectory stability was evaluated using RMSD, root-mean-square fluctuations (RMSF), and radius of gyration (Rg) calculations.

## 3. Results

### 3.1. Evaluating HPgV Homology Structures

HCV direct-acting antivirals (DAAs) mainly target NS3/4A, NS5A, and NS5B [36]. Therefore, we first asked whether the corresponding HPgV non-structural proteins are sufficiently related to HCV to support comparative modeling. A consensus sequence for the HPgV polyprotein was annotated for all HPgV-NS proteins which were submitted to SWISS-MODEL for homology modeling. Only NS3/4A and NS5B models passed the predetermined threshold (GMQE ≥ 0.7), and thus the subsequent work focuses on only these two proteins. The HPgV NS3/4A homology model was based off the HCV NS3/4A crystal structure (PDB ID: 4A92) [24], and the HPgV homology model on the HCV NS5B structure (PDB ID: 1YUY) [25]. Primary protein sequences showed 43% amino acid (AA) identity and 55% similarity between the HPgV NS3 consensus sequence and HCV NS3 (PDB ID: 4A92) across a 606-residue alignment (HCV residues 46–638; HPgV residues 10–597; 5% gaps), corresponding to 91% sequence coverage of the HCV NS3 and 97% coverage of the HPgV NS3 consensus sequence. The NS5B HPgV consensus sequence shares 34% AA identity and 50% similarity with the HCV NS5B (PDB ID: 1YUY) across a 506-residue alignment (HCV residues 1–470; HPgV residues 1–478; 8% gaps), corresponding to 89% coverage of HCV and 100% coverage of the HPgV protein sequence. The HPgV NS3/4A and NS5B structures had GMQEs of 0.75 and 0.70, respectively. GMQE reflects the suitability of the chosen template and does not directly measure the biological accuracy of the resulting model. Ramachandran plots were used to analyze the energetic favorability of backbone dihedral angles (Supplementary Materials). The NS3/4A homology model covered 619 residues (98.9%) of the template protein, of which 92.5% fell into favored regions of the Ramachandran plot. The NS5B model covered 478 residues (99.6%) of the template protein yielding 90.2% of residues in favored regions.

**Figure 2:**
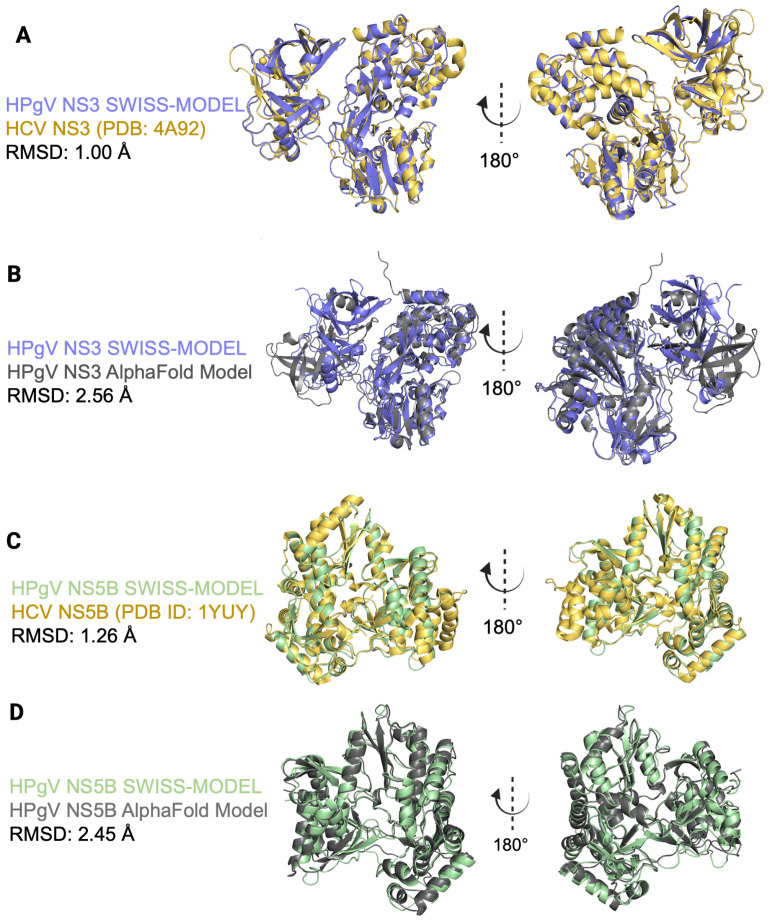
HPgV NS3 and NS5B Homology Models. (**A**) HCV NS3 (PDB: 4A92, yellow) and HPgV (purple) NS3 subunits aligned. (**B**) HPgV NS3 SWISS-MODEL homology model (purple) aligned to AlphaFold3 predicted HPgV NS3 (gray). (**C**) HCV NS5B (PDB: 1YUY, yellow) and HPgV (purple) NS3 subunits aligned. (**D**) HPgV NS5B SWISS-MODEL homology model (purple) aligned to AlphaFold3 predicted HPgV NS3 (gray).

Next, we compared the structural differences between HCV and HPgV by aligning and visually inspecting the secondary structures. The NS3/4A structures were highly similar, with the main differences occurring in the loops connecting alpha helices and beta sheets (Figure 2A). The RMSD between all backbone atoms of the HPgV NS3/4A homology model and the HCV NS3/4A crystal structure was 1.00 Å (Figure 2A). The all-atom RMSD between the HPgV NS5B homology model and the HCV NS5B crystal structure was 1.26 Å (Figure 2C). Additionally, we compared the HPgV homology models from SWISS-MODEL to AlphaFold2 predicted structures (Figure 2B,D); the NS3/4A homology models had an RMSD of 2.56 Å and the NS5B model RMSD was 2.45 Å. Together, these results indicate the HPgV SWISS-MODEL predicted structures for NS3/4A and NS5B are stable and can be used for molecular docking.

### 3.2. Structure-Based Virtual Screening

Next, we predicted the binding affinity of all FDA-approved small molecules (*n* = 2203) to determine the active site similarity between HCV and the modeled HPgV. Binding scores reflect predicted binding affinity within the defined active site; negative binding affinity scores indicate a stronger interaction, while scores of 0 or higher are predicted to have no interaction. Across all small molecules, the HCV and HPgV NS3/4A SWISS-MODEL protein had moderate but significant correlation in docking scores (r = 0.46, 95% CI [0.43, 0.49]; *p* < 0.001). The HPgV NS3/4A AlphaFold model was also significantly correlated with HCV NS3 (r = 0.71, 95% CI [0.69, 0.73]; *p* < 0.001), indicating that the modeled HPgV NS3 configurations would likely bind to similar compounds as HCV NS3.

NS5B proteins had a moderately strong correlation between both HCV and HPgV SWISS-MODEL docking affinities (r = 0.76, 95% CI [0.75, 0.78]; *p* < 0.001) and HCV and HPgV AlphaFold affinities (r = 0.68, 95% CI [0.67, 0.70]; *p* < 0.001), which suggests greater conservation in the modeled polymerase pocket relative to NS3/4A. The affinities for the top 10 docked small molecules across all drug classes are shown in Supplemental Tables S3 and S4. Only one HCV DAA (Paritaprevir) was identified as a top docking target which reflects that docking affinity is determined by the modeled active site microenvironment and does not incorporate broader structural features, protein dynamics, or conformational states relevant to true inhibitor activity. Interestingly, out of the top 10 predicted binding partners identified with NS3 proteins, 7 of 10 ligands were shared between HCV and HPgV SWISS-MODEL (70% overlap), whereas NS5B showed no overlap in top binders. This difference is consistent with the higher primary sequence similarity and template-based modeling confidence for NS3/4A described above and suggests that NS3 may represent the more structurally conserved target for potential therapeutic repurposing.

The predicted docking scores for HCV DAAs against the homology modeled HPgV NS3 are shown in Table 2. For NS3/4A, these included Glecaprevir, Paripatrevir, Simeprevir, Voxilaprevir, Grazoprevir, Boceprevir, and Telaprevir. Both HCV and HPgV NS3/4A proteins were favorably predicted to bind with all 5 DAAs, with modest improved docking for HCV relative to HPgV (HCV: −7.5 to −12.5 kcal/mol; HPgV SWISS-MODEL: −7.4 to −11.5 kcal/mol, HPgV AlphaFold: −6.2 to −8.9 kcal/mol). HCV NS5B DAAs (Dasabuvir, Sofosbuvir) showed a similar pattern (Table 3). Both DAAs bound favorably to the defined HCV and HPgV NS5B active sites, with modestly more favorable binding to the HCV cognate than to HPgV (HCV: −7.5 to −11.5 kcal/mol; HPgV SWISS-MODEL: −7.2 to −8.3 kcal/mol, AlphaFold: −7.0 to −8.3 kcal/mol). Because we were unable to generate a stable HPgV NS5A model, NS5A inhibitors (Ledipasvir, Pibrentasvir, Elbasvir, Velpatasvir) were included only as part of the broad FDA-approved ligand screen, but they do not have known or predicted binding sites in NS3/4A or NS5B. Therefore, while docking scores are reported for completeness (Table S5), they were not interpreted in comparative analyses.

**Figure 3:**
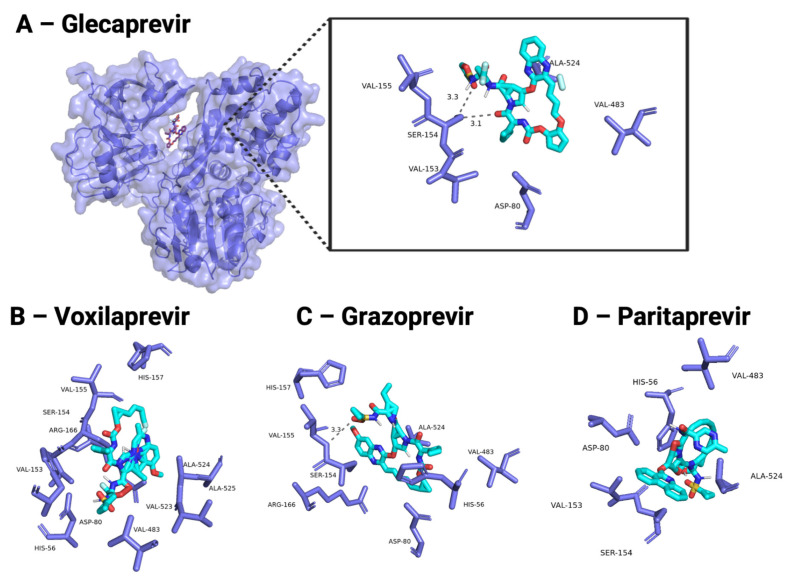
Top docked compounds against HPgV NS3. (**A**) The HPgV NS3 homology model (purple cartoon and surface) with Glecaprevir docked (shown in stick representation with carbon atoms in light blue, hydrogen atoms in light grey, nitrogen atoms in dark blue, oxygen atoms in red, and sulfur atoms in yellow). The zoomed-in pose shows the hydrogen bonds (gray dashes) and the labeled residues involved in binding (purple). (**B**) Voxilaprevir, (**C**) Grazoprevir, and (**D**) Paritaprevir, docked poses.

**Figure 4:**
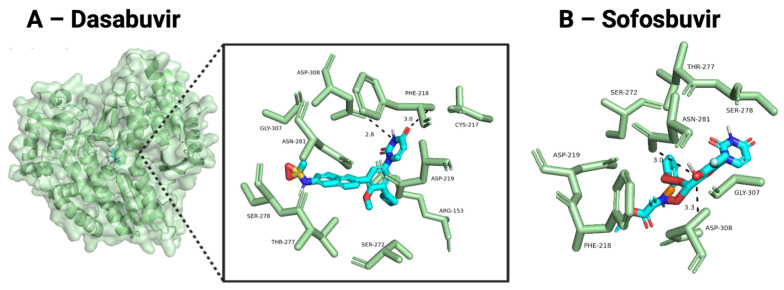
Top docked compounds against HPgV NS5B. (**A**) The HPgV NS5B homology model (green cartoon and surface) with Dasabuvir docked (shown in stick representation with carbon atoms in light blue, hydrogen atoms in light grey, nitrogen atoms in dark blue, oxygen atoms in red, and sulfur atoms in yellow). The zoomed-in pose shows the hydrogen bonds (gray dashes) and the labeled residues involved in binding (green). (**B**) active Sofosbuvir docked poses.

The protein–ligand interactions between HCV antivirals and HPgV NS3 and NS5B SWISS-MODEL subunits are visualized in Figure 3 and Figure 4. Ligands docked against HPgV NS3/4A were assessed for interaction with the catalytic triad (HIS56, ASP79, SER137) essential for enzyme activity. Glecaprevir, an NS3/4A inhibitor, had the best affinity when docked against HPgV NS3 (−11.5 kcal/mol), likely due to the two hydrogen bonds formed with SER154 (Figure 3A). Voxilaprevir, Grazoprevir, and Paritaprevir, all NS3/4A inhibitors, had hydrophobic interactions with HIS56 (Figure 3B–D). These ligands also interacted with ASP80, VAL153, SER154, VAL155, VAL483, and ALA524 of HPgV NS3.

Ligands docked against NS5B were assessed for interaction with ASP219, ASP226, or the GDD motif (residues 307–309) that binds metal cations and is essential for viral RNA synthesis. Dasabuvir had relatively low predicted binding affinities against HPgV NS5B (−8.5 kcal/mol) compared to HCV NS5B (−11.5 kcal/mol). Predicted affinities were similar for Sofosbuvir docked against HPgV (−7.2 kcal/mol) and HCV (−7.5 kcal/mol). However, both Dasabuvir and Sofosbuvir had predicted interactions with ASP219 and hydrogen bonds with ASP308. Other residues predicted to be important for HPgV NS5B binding were PHE218, SER272, THR277, SER278, and GLY307.

### 3.3. Molecular Dynamics Simulations

The apo HPgV NS3/4A and NS5B structures were used in 100 ns molecular dynamics (MD) simulations to examine stability, conformational changes, and binding site flexibility. The HPgV NS3/4A backbone fluctuated around a mean RMSD of 4.5 ± 1.1 Å with mean Rg stabilized around 27.0 ± 0.20 Å. The HPgV NS5B model had a mean RMSD of 4.2 ± 0.60 Å and Rg of 22.8 ± 0.42 Å.

**Figure 5:**
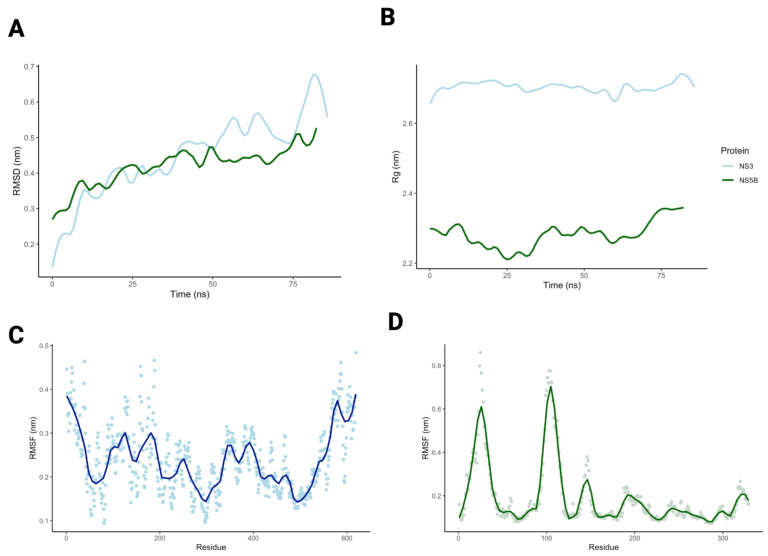
Molecular dynamics results. (**A**) The backbone RMSD of the HPgV NS3 (blue) and NS5B (green) proteins over the 100 ns simulation. (**B**) The radius of gyration of the HPgV NS3 (blue) and NS5B (green) proteins tracked throughout the trajectory to evaluate protein compactness. (**C**) NS3 and (**D**) NS5B per-residue RMSF for Cα atoms. Trajectory lines were smoothed in each graph using LOESS to highlight the overall trend.

RMSF analysis revealed that the NS3/4A structured core remained stable throughout the simulation, with most residues fluctuating between 1.5 and 2.5 Å (Figure 5C). Higher flexibility was observed at the N- and C-termini (RMSF 3.5–4.0 Å), consistent with their expected disorder. Several loop regions (residues ~120–150, ~220–260, ~320–360, and ~400–440) exhibited moderate flexibility (RMSF~3.0 Å). The catalytic triad remained stable (RMSF < 2.0 Å). The NS5B model had high flexibility (RMSF 6.0–8.0 Å) in loop regions connecting alpha helices and beta sheets (residues ~20–30, ~90–125). The residues important to ligand binding, the GDD motif, had minimal fluctuations (RMSF < 2.0 Å). Together, the low RMSD and stable catalytic triad of HPgV NS3/4A over 100 ns indicate that the modeled NS3/4A active site remains well-ordered under simulation. This structural stability, combined with the similarity of predicted NS3 docking preferences between HPgV and HCV, supports NS3/4A as the most plausible candidate for experimental follow-up with HCV NS3/4A inhibitors.

## 4. Discussion

To our knowledge, this is the first study of protein structures of druggable targets for HPgV. Our homology models for the HPgV NS3/4A serine protease and the NS5B RNA-dependent RNA polymerase (RdRp) built on experimentally derived structures suggest that HPgV will respond to HCV DAAs, particularly those that target the HCV NS3 protein. Despite their sequence differences, the predicted structures of HPgV NS3/4A and NS5B aligned well to HCV experimental structures.

Moreover, de novo protein folding by AlphaFold obtained structures that differed by 2.56 and 2.45 Å from the HCV modeled NS3/4A and NS5B proteins, respectively. While SWISS-MODEL necessarily uses the closest structural homolog (HCV) as a fold scaffold, the independent AlphaFold predictions converged on a very similar topology. This convergence demonstrates that the backbone arrangement proposed by SWISS-MODEL is not an artifact of template imposition but rather a fold that emerges from the HPgV sequence itself. In turn, the similarities across AlphaFold docking increase confidence that docking HCV DAAs into these modeled active sites is not purely speculative: although docking reflects only pocket geometry, the agreement between template-based and de novo models supports that the binding pockets themselves are plausibly represented. While AlphaFold performs de novo protein modeling, it should be noted that the algorithm is highly influenced by PDB structures included in model training.

Both NS3 and NS5B showed high affinity for HCV DAAs in their binding pockets. While molecular docking cannot predict the ability of ligands to access the binding pocket, it is clear that the modeled 3-dimensional structures were stable when using the HCV backbone. Most HCV-NS3/4A targeting drugs function by binding to the NS3/4A serine protease active site preventing the cleavage of the HCV polyprotein needed for viral replication [10,11,16]. We have shown that several HCV antivirals, including Glecaprevir, Paripatrevir, Simeprevir Voxilaprevir and Grazoprevir, interacted with one or more residues of the key AA involved in the catalytic triad of HPgV NS3/4A when docked, indicating potential therapeutic potential. Boceprevir and Telaprevir exhibited notably worse predicted docking affinity against both HPgV and HCV proteins compared to Glecaprevir despite having similar binding mechanisms. Boceprevir and Telaprevir are first-generation DAA’s that have been discontinued for HCV use and did not inhibit HPgV in cell culture [36]. While docking for newer generations of HPgV NS3/4A inhibitors showed promising results, there is a chance the older DAAs would not inhibit HPgV in vitro.

DAAs targeting HCV NS5B bind to the active site palm domain that contains the GDD motif. Sofosbuvir is one of the most potent HCV NS5B inhibitors due to its broad application across HCV genotypes; however, we found that this compound had the least predicted binding affinity of all HCV antivirals tested. Sofosbuvir is a prodrug that is metabolized in the liver prior to NS5B binding [37]. We docked both the inactive and active metabolized form of Sofosbuvir with minimal improvement in binding affinity when the active compound was used for both the HCV and HPgV NS5B subunits. The active form of sofosbuvir inhibits HCV by promoting chain termination, a process that involves many conformational changes that are not fully modeled during docking and, therefore, result in suboptimal binding scores.

There are limitations to structure-based docking methods, reflected by predicted NS5A inhibitor binding to NS3/4A or NS5B. Both the NS3/4A and NS5B active sites have a large substrate groove with hydrophobic pockets. During docking, the NS5A inhibitors are forced into the active site and form non-specific contacts between hydrophobic surfaces. The docking algorithm rewards this false interaction and artificially inflates the predicted affinity between NS5A inhibitors and NS3 or NS5B, even though true biochemical binding would be weak. Therefore, we began assessing conformational dynamics of NS3/4A and NS5B with molecular dynamics simulations.

Our molecular dynamics analysis showed that the HPgV NS3/4A SWISS-MODEL is not only very similar to the de novo AlphaFold model, but it is predominantly stable with minimal fluctuations restricted to the N and C termini, which do not participate in catalytic activity. Overall, the distribution of flexibility across HPgV NS3/4A aligns with known structural and functional expectations and supports the suitability of the model for future mechanistic analyses.

Similarly, the HPgV NS5B polymerase maintained a stable global structure throughout the molecular dynamic trajectory. These results suggest that the overall fold of the RdRp remained compact and did not undergo large-scale conformational rearrangements. Thus again, these results indicate that the NS5B model behaves as a structurally stable polymerase with expected loop mobility.

Our findings may have important clinical implications. There is established evidence that HCV and HPgV infections often coexist in humans [2,38]. Epidemiologic studies have also shown an association between HCV infection and PD [39,40,41,42], and a decreased risk of PD in patients with Hepatitis C treated with interferon-based antivirals [43,44]. Since HCV was not found in the brains of PD patients [9], it is therefore possible that the associations of HCV and PD may be due to co-infection, possibly with a virus such as HPgV, and that the effect of HCV antivirals on the risk of PD was caused by their off-target effects against HPgV. Indeed, recent studies showed decreased HPgV viremia in Hepatitis C patients treated with anti-HCV medications, which included combination therapy of sofosbuvir/ledipasvir (Harvoni) and sofosbuvir/velpatasvir (Ecplusa), both targeting NS5B and NS5A proteins, and pegylated interferon [15,45]. The various drug combinations suggested that the decrease in HPgV viral load in blood was caused by the NS5B effect of sofosbuvir, whereas glecaprevir/pibrentasvir (Mavyret) targeting the NS3/4A and NS5A proteins had no effect.

There are several limitations to this study, given these are in silico predictions from HPgV consensus sequences with no experimental structures or binding data. Thus, the fine details of the HPgV structures here should be interpreted with caution. Protein–ligand molecular dynamics simulations were out of the scope of this work, but future work will evaluate the ability of HCV DAAs to inhibit viral replication of HPgV in vitro.

## 5. Conclusions

We created homology models for two key HPgV proteins lacking experimental structures, but which have high sequence and predicted functional similarities to HCV proteins which are well understood as an important first step for future drug development. Our data strongly suggest that multiple commercially available antiviral medications for HCV can also be used to target HPgV. Together with clinical studies showing decreased HPgV viremia after treatment with a compound targeting the HCV NS5B protein, these results are tantalizing and open new avenues of investigations on potential repurposing of anti-HCV medications for PD.

## Figures and Tables

**Table 1:** FDA-Approved HCV direct-acting antivirals.

Trade Name	Small Molecule Combinations	HCV Protein Target(s)		
		NS3	NS5A	NS5B
Harvoni	Ledipasvir/Sofosbuvir		X	X
Zepatier	Grazoprevir/Elbasvir	X	X	
Epclusa	Velpatasvir/Sofosbuvir		X	X
Vosevi	Voxilaprevir/Velpatasvir/Sofosbuvir	X	X	X
Mavyret	Glecaprevir/Pibrentasvir	X	X	

**Table 2:** Docking Affinity (kcal/mol) of approved direct-acting antivirals for Hepatitis C Virus against HPgV NS3/4A SWISS-MODEL homology model, AlphaFold2 model, and HCV experimental structure (PDB: 4A92).

Drug	HCV Protein Target	Docking Affinity (kcal/mol)		
		HPgV NS3/4A SWISS-MODEL	HPgV NS3/4A AlphaFold Model	HCV NS3/4A (PDB ID: 4A92)
Glecaprevir	NS3/4A	−11.5	−8.9	−12.5
Paritaprevir	NS3/4A	−11.4	−8.6	−12.4
Simeprevir	NS3/4A	−10.7	−8.6	−11.8
Voxilaprevir	NS3/4A	−10.6	−8.9	−11.8
Grazoprevir	NS3/4A	−9.8	−7.6	−11.1
Telaprevir	NS3/4A	−7.6	−6.2	−8.4
Boceprevir	NS3/4A	−7.4	−6.3	−7.5

**Table 3:** Docking affinity (kcal/mol) of approved direct-acting antivirals for Hepatitis C Virus against HPgV NS5B SWISS-MODEL homology model, AlphaFold2 model, and HCV experimental structure (PDB: 1YUY).

Drug	HCV Protein Target	Docking Affinity (kcal/mol)		
		HPgV NS5B SWISS-MODEL	HPgV NS5B AlphaFold Model	HCV NS5B (PDB ID: 1YUY)
Dasabuvir	NS5B	−8.5	−8.3	−11.5
Sofosbuvir	NS5B	−7.2	−7.0	−7.5

## Data Availability

Data is available from the corresponding author upon reasonable request.

## Supplementary Materials

The following supporting information can be downloaded at https://www.mdpi.com/article/10.3390/v18020261/s1, FASTA sequences; Table S1: Confidence Scores for AlphaFold Models; Table S2: Human Pegivirus Non-Structural Protein SwissModel Homology Model Evaluation; Table S3: Affinity (kcal/mol) of top-predicted FDA-approved compounds against the NS3 HPgV NS3 homology model and HCV NS3 (PDB: 4A92); Table S4: Affinity (kcal/mol) of top-predicted FDA-approved compounds against the HPgV NS5B homology model and HCV NS5B (PDB ID: 1YUY); Table S5: Docking affinity (kcal/mol) of approved HCV NS5A inhibitors against HPgV NS3 SWISS-MODEL homology model, AlphaFold2 model, and HCV experimental structure (PDB: 4A92) and HPgV NS5B SWISS-MODEL homology model, AlphaFold2 model, and HCV experimental structure (PDB: 1YUY); Figure S1: NCBI Blast alignment of the HCV NS3 consensus sequence.
