# Supplementary material for: In Silico Characterization of Two Human Pegivirus Proteins Highlights Similarities with Hepatitis C Virus and Possible Therapeutic Repurposing
Source: Viruses. 2026 Feb 19;18(2):261. doi: 10.3390/v18020261 (PMC12945168; doi:10.3390/v18020261)
Supplement: Supplementary file 1 [file viruses-18-00261-s001.zip › viruses-4087931-supplementary.pdf]

viruses

MDPI

Supplementary Information

FASTA Sequences

HCV NS3 (PDB: 4A92)

GSITAYSQQTRGLLGCIITSLTGRDKNQVEGEVQVVSTATQSFLATCVNGVCWTVYHGAG-
SKTLAGPKGPITQMYTNVDQDLVGWQAPPGARSLTPCTCGSSDLYLVTRHADVIPVRRRGDSRGSLLSPRPVSY
LKGSSGGPLLCPSGHAVGIFRAAVCTRGVAKAVDFVPVESMETTMRSPVFTDNSSPPAV-
PQSFQVAHLHAPTGSGKSTKVPAAYAAQGYKVLVLNPSVAATLGFGAYMSKAHGIDPNIRTGVRTITTGAPV
TYSTYGKFLADGGCSGGAYDIIICDECHSTDSTTILGIGTVLDQAETAGARLVVLA-
TATPPGSVTVPHPNIEEVALSNTGEIPFYGKAIPIEAIRGGRHLIFCHSKKKCDELAAKLSGLGINAVAYYRGLD
VSVIPTIGDVVVVATDALM-
TGYTGDFDSVIDCNTCVTQTVDFSLDPTFTIETTTVPQDAVSRSQRRGRTGRGRRGIYRFVTPGERPSGMFDSSV
LCECYDAGCAWYELTPAETSVRLRAYLNTPGLPVCQDHLEFWESVFTGLTHID-
AHFLSQTKQAGDNFPYLVAYQATVCARAQAPPPSWDQMWKCLIRLKPTLHGPTPLLYRLGAVQNEVTLTHPI
TKYIMACMSADLAVVL

HCV NS5B (PDB: 1YUY)

SMSYSWTGALITPCSPEEEKLPINPLSNSLLRYHNKVYC-
TTSKSASLRAKKVTFDRMQVLDAYYDSVLKDIKLAASKVSARLLTLEEACQLTPPHSARSKYGFGAKEVRSLSG
RAVNHIKSVWKDLLEDSQTPIPTTIMAKNEVFCVDPTKGGKKAARLIVYPDLGVRVCEKMAL-
YDVTQKLPQAVMGASYGFQYSPAQRVEFLLKAWAEKKDPMGFSYDTRCFDSTVTERDIRTEESIYQACSLPEE
ARTAIHSLTERLYVGGPMFNSKGQSCGYRRCRASGVLTTSMGNTITCYVKALAACKAA-
GIVAPTMLVCGDDLVVISESQGTEEDERNLRAFTEAMTRYSAPPGDPPRPEYDLELITSCSSNVSVALGPQGRRR
YYLTRDPTTPIARAAWETVRHSPVNSWLGNIIQYAPTIWVRMVLMTHFFSILMAQD-
TLDQNLNFEMYGSVYSVSPLDLPAIIERLHGLDAFSLHTYTPHELTRVASALRKLGAPPLRAWKSRARAVRASL
ISRGGRAAVCGRYLFNWAVKTKLKLTPLPEARLLDLSSWFTVGAGGGDIYHSVSRARPR

HPgV NS3 – Consensus

APVVIRRCGKGFLGVTKAALTGRDPDLHPGNVMVLG-
TATSRSMGTCLNGLLFTTFHGASSRTIATPVGALNPRWWSASDDVTVYPLPDGATSLTPCTCQAESCWVIRSD
GALCHGLSKGDKVELDVAMEVSDFRGSSGSPVLCDEGHAVGMLVSVLHSGGRVTAARFTR-
PWTQVPTDAKTTTEPPPVPAKGVFKEAPLFMPTGAGKSTRVPLEYDNMGHKVLILNPSVATVRAMGPYMERL
AGKHPSIYCGHDTTAFTRITDSPLTYSTYGRFLANPRQMLRGVSV-
VICDECHSHDSTVLLGIGRVRELARGCGVQLVLYATATPPGSPMTQHPSIIETKLDVGEIPFYGHGIPLERMRTG
RHLVFCHSKAECERLAGQFSARGVNAIAYYRGKDSSIIKDGDLVVCAT-
DALSTGYTGNFDSVTDCGLVVEEVVEVTLDPTITISLRTVPASAELSMQRRGRTGRGRSGRYYYAGVGKAPAGV
VRSGPVWSAVEAGVTWYGMEPDLTANLLRLYDDCPYTAAVAADIGEAAVFFSGLAPLRM-
HPDVSWAKVRGVNWPLLVGVQRTMCRETLSPGPSDDPQWAGLKGPNPVPLLLRWGNDLPSKVAGHHIVDD
LVRRLGVAEGYVRC

HPgV NS5B - Consensus

SMSYSWSGVPLTRATPAKPPVVRPVGSLLVADTT-
KVYYVTNPDNVGRRVDKVTFWRAPRVHDKFLVDSIERARRAAQACLSMGYTYEEAIRTVRPHAAMGWGSKVS
VKDLATPAGKMAVHDRLQEILEGTPVPFTLTVKKEVFFKDRKEEKAPRLIVFPPLDFRI-
AEKLILGDPGRVAKAVLGGAYAFQYTPNQRVKEMLKLWESKKTPCAICVDATCFDSSITEEDVALETELYALA

SDHPEWVRALGKYYASGTMVTPEGVPVGERYCRSSGVLTTSASNCLTCYIKVKAACERVGLK-
NVSLLIAGDDCLIICERPVCDPSDALGRALASYGYACEPSYHASLDTAPFCSTWLAECNADGKRHFFLTTDFRR
PLARMSSEYSDPMASAIGYILLY-
PWHPITRWVIIPHVLTCAFRGGGTPSDPVWCQVHGNYYKFPLDKLPNIIVALHGPAALRVTADTTKTKMEAG
KVLSDLKLPGLA

Table S1. Confidence Scores for AlphaFold Models.

HPgV Subunit	Per-Atom confidence estimate (Overall score)	Predicted Template Modeling (pTM)	Interface Predicted Temple Modeling (iPTM)	Chain-Pair Predicted Aligned-Error (PAE)
NS3	0.80	0.79	0.79	0.76
NS5B	0.95	0.89	0.89	0.76

Table S2. Human Pegivirus Non-Structural Protein SwissModel Homology Model Evaluation.

Protein	PDB ID Modeled After	GMQE	QmeanDisCo
**NS3**	4A92	0.75	0.72 +/- 0.05
**NS5B**	1YUY	0.75	0.70 +/- 0.05

PDB: Protein Database; GMQE: Global model quality estimate.

Table S3. Affinity (kcal/mol) of top-predicted FDA-approved compounds against the NS3 HPgV NS3 homology model and HCV NS3 (PDB: 4A92).

Drug	Disease	Mechanism of Action	HPgV NS3 SWISS-MODEL		HPgV NS3 AlphaFold		HCV NS3 (PDB ID: 4A92)	
			Rank	Affinity	Rank	Affinity	Rank	Affinity
Rifabutin	Bacterial Infection	Inhibits DNA-dependent RNA-polymerase	1	-12.7	3	-10.6	1	-14.1
Lurbinectedin	Small-cell Lung Cancer	DNA alkylating agent	2	-12.0	6	-10.0	5	-13.5
Sirolimus or Rapamycin	Immunosuppressant	mTOR inhibitor	3	-12.0	24	-9.5	6	-13.5
Rifaximin	Bacterial Infection	Inhibits DNA-dependent RNA-polymerase	4	-11.9	7	-10.0	7	-13.4
Trabectedin	Soft Tissue Sarcoma	DNA alkylating agent	5	-11.7	12	-9.7	4	-13.6
Omaveloxolone	Friedreich's ataxia	Activates Nrf2, inhibits NF-KB	6	-11.6	2	-10.6	NA	NA
Eribulin	Metastatic breast cancer	Microtubule inhibitor	7	-11.5	14	-9.6	10	-13.1
Glecaprevir	HCV	Inhibits NS3/4A protease	8	-11.5	45	-8.9	11	-12.5
Dihydroergotamine	Migraines	5-hydroxytryptamine 1b agonist	9	-11.4	4	-10.5	9	-13.1
Paritaprevir	HCV	Inhibits NS3/4A protease	10	-11.4	80	-8.6	30	-12.4
Ergotamine	Migraines	Vasoconstrictor, Ergot alkaloid	12	-11.3	1	-11.2	3	-13.6

Dutasteride	Enlarged prostate	5-alpha reductase inhibitor	43	-10.4	34	-9.1	8	-13.2
Dihydroergocristine	Peripheral vascular disease	Adrenergic Receptor Antagonist	15	-11.2	13	-9.6	2	-13.6

NA: Not Available.

Table S4. Affinity (kcal/mol) of top-predicted FDA-approved compounds against the HPgV NS5B homology model and HCV NS5B (PDB ID: 1YUY).

			HPgV NS5B SWISS-MODEL		HPgV NS5B Alpha-Fold		HCV NS5B (PDB ID: 1YUY)	
**Drug**	**Disease**	**Mechanism of Action**	**Rank**	**Affinity**	**Rank**	**Affinity**	**Rank**	**Affinity**
Eslicarbazepine acetate	Epilepsy	Inhibition voltage gated NA+ channels	1	-14.8	562	-7.3	439	-10.5
Vigabatrin	Epilepsy	GABA inhibitor	2	-14.4	2145	-4.0	2160	-4.9
Linagliptin	Type 2 Diabetes	DPP-4 Inhibitor	3	-14.3	340	-7.8	490	-10.3
Prednisone	Corticosteroid	Glucocorticoid receptor agonist	4	-14.2	285	-8.0	174	-12.5
Ecamsule	Sunburn prevention	Prevents UV ray absorption	5	-14.2	190	-8.3	29	-13.6
Etacrynic acid	Diuretic	Inhibits symport of Na, K, and Cl	6	-14.2	1459	-5.7	1567	-7.2
Oxycodone	Opioid	Mu Opioid receptor agonist	7	-13.6	349	-7.8	118	-12.5
Sodium tetradecyl sulfate	Varicose veins	Endothelial cell toxin	8	-13.4	NA	NA	NA	NA
Cloxacillin	Antibiotic	Inhibits penicillin-binding proteins	9	-13.3	662	-7.1	700	-9.5
Nevirapine	HIV	Inhibits RNA-dependent and DNA-dependent polymerases	10	-13.2	772	-6.9	713	-9.5
Omaveloxolone	Bacterial Infection	Inhibits DNA-dependent RNA-polymerase	989	-8.3	16	-10.3	1	-16.2
Dihydroergotamine	Migraines	Vasoconstrictor, Ergot alkaloid	1176	-7.9	30	-10.0	2	-15.3
Ergotamine	Migraines	Vasoconstrictor, Ergot alkaloid	1754	-6.5	20	-10.2	3	-15.3
Vincristine	Soft Tissue Sarcoma	DNA alkylating agent	970	-8.3	42	-9.7	4	-14.8
Drospirenone	Small-cell Lung Cancer	DNA alkylating agent	435	-9.8	66	-9.2	5	-14.7
Dihydroergocristine	Peripheral vascular disease	Ergot alkaloid	260	-10.4	15	-10.4	6	-14.6
Betulinic Acid	Immunosuppressant	mTOR inhibitor	NA	NA	52	-9.5	7	-14.6
Glycyrrhizic acid	Liver disease, ulcers	inhibits the enzyme	1982	-5.6	35	-9.9	8	-14.6

Sirolimus or Rapamycin	Immunosuppressant	mTOR inhibitor	1367	-7.4	3	-11.5	9	-14.6
Tubocurarine	Neuromuscular blocker	Competes with acetylcholine for binding	1471	-7.2	19	-10.3	10	-14.5

NA: Not Available

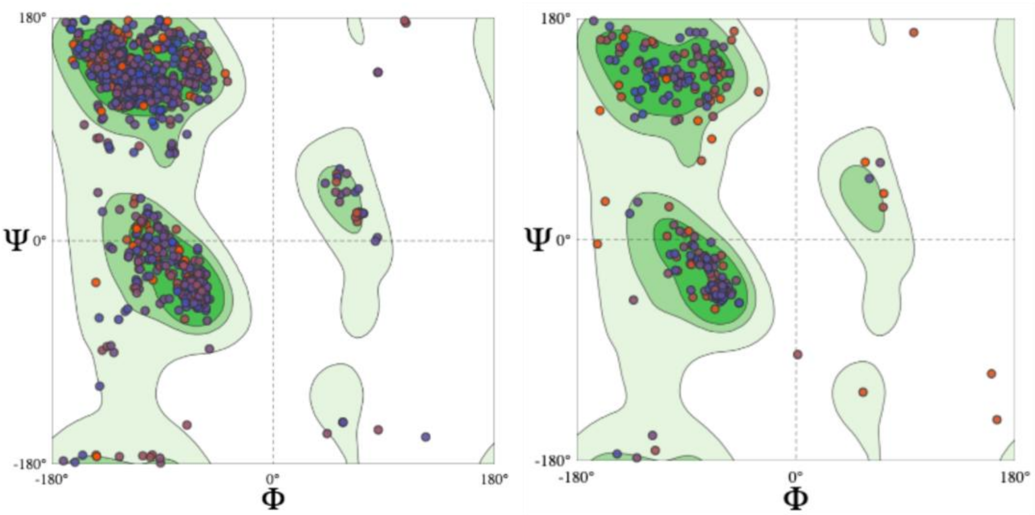

(a) (b)

Figure S1. A) NCBI Blast alignment of the HCV NS3 consensus sequence (black) to the HPgV NS53 consensus sequence (red). B) Ramachandran plot for the HPgV SwissModel Predicted Homology Model for A) NS3 and B) NS5B.
